# Nucleation of Poly(lactide) Partially Wet Droplets in Ternary Blends with Poly(butylene succinate) and Poly(ε-caprolactone)

**DOI:** 10.1021/acs.macromol.9b02295

**Published:** 2020-02-25

**Authors:** Seif Eddine Fenni, Jun Wang, Nacerddine Haddaoui, Basil D. Favis, Alejandro J. Müller, Dario Cavallo

**Affiliations:** † Department of Chemistry and Industrial Chemistry, University of Genova, via Dodecaneso, 31, 16146 Genova, Italy; ‡ Laboratory of Physical-Chemistry of High Polymers (LPCHP), Faculty of Technology, University of Ferhat ABBAS Sétif-1, 19000 Sétif, Algeria; § CREPEC, Department of Chemical Engineering, École Polytechnique de Montréal, Montréal, Québec H3C3A7, Canada; ∥ POLYMAT and Polymer Science and Technology Department, Faculty of Chemistry, University of the Basque Country UPV/EHU, Paseo Manuel de Lardizábal 3, 20018 Donostia-San Sebastián, Spain; ⊥ IKERBASQUE, Basque Foundation for Science, 48013 Bilbao, Spain

## Abstract

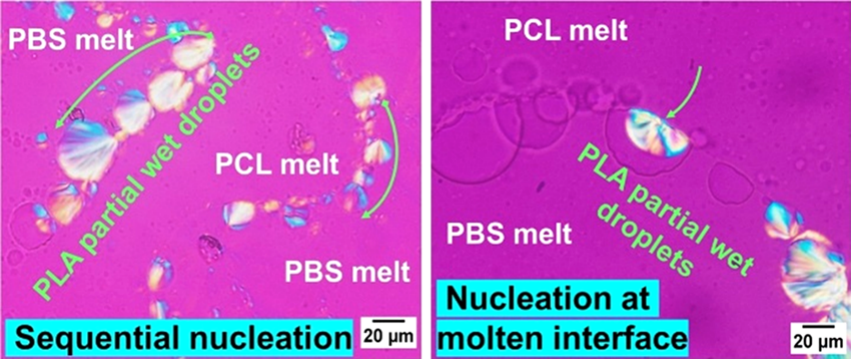

This work presents the first investigation on the crystallization behavior of partially wet droplets in immiscible ternary blends. Poly(lactide), poly(ε-caprolactone), and poly(butylene succinate) (PLA, PCL, and PBS, respectively) were melt blended in a 10/45/45 weight ratio to produce a “partial wetting” morphology with droplets of the PLA minor phase located at the interface between the other two major components. The crystallization process of the higher melting PLA droplets was studied by polarized light optical microscopy, while the other components remain in the molten state. We found that neighboring partially wet droplets nucleate in close sequence. This is unexpected since partially wet droplets display points of three-phase contact and, hence, should not touch each other. Moreover, the onset of poly(lactide) crystallization is frequently observed at the interface with molten PCL or PBS, with a significant preference for the former polymer. The observed sequential droplet-to-droplet crystallization is attributed to the weak partial wetting behavior of the PCL/PLA/PBS ternary system. In fact, the contact between the interfacially confined droplets during crystallization due to their mobility can lead to a transition from a partial to a completely wet state, with the formation of thin continuous layers bridging larger partially wet droplets. This allows crystallization to spread sequentially between neighboring domains. Using a simple heterogeneous nucleation model, it is shown that the nucleation of PLA on either PCL or PBS melts is energetically feasible. This study establishes a clear relationship between the unique partial wetting morphology of ternary blends and the nucleation of the minor component, paving the way to the understanding and control of crystallization in multiphasic polymer blends for advanced applications.

## 1 Introduction

Polymer blending is an extensively used method for tailoring and/or modifying the properties of polymers. Until recently, binary blends, i.e., composed of two polymers, have mainly been considered. The most frequently encountered phase-separated morphologies of immiscible binary blends are the droplet/matrix and co-continuous. Several parameters play a role in determining the obtained morphology, both related to the polymer themselves (i.e., composition, viscosity ratio, and interfacial tension) or to processing (thermomechanical history of the sample).^1−3^ Recently, considerable attention has been paid to multicomponent polymer blends, comprising at least three immiscible polymers. Such systems can result in a set of entirely new materials, such as high-performance bioplastics,^4,5^ hierarchically porous polymers,^6,7^ and conductive polymer blends with ultra-low percolation thresholds.^8,9^ They can even be used as an approach to recycling co-mingled waste plastics.^10^

A variety of phase morphologies can be obtained, in multiphase polymer blends, which offers the possibility to tune the properties of the resulting material.^11−26^ For example, in ternary blends composed of two principal phases (A and B) and one minor phase (C), four types of morphologies are possible.^14^ Phase C may be completely engulfed by either phase A or phase B, or it can form a thin layer completely wetting the A/B interface. These three scenarios are called complete wetting morphologies, where one of the phases fully separates the other two.^9,27,28^ In the fourth case, phase C is present as droplets at the A/B interface, demonstrating a partial wetting morphology.^14,19^ Ternary blends of 45/10/45 polylactide/ethylene methacrylate/polyamide 11 (PLA/EMA/PA11) and 50/5/45 polycaprolactone/polybutylene succinate/polylactide (PCL/PBS/PLA) are among the systems that have been reported to show partially wet interfaces by middle phase droplets.^23,25^

The understanding and control of ternary blend morphology are of importance since it can completely alter the final mechanical performance of the material, resulting in highly synergistic effects in certain cases.^11−13,29^ For example, brittle binary polymer blends comprising PLA can be efficiently toughened by adding a suitable third component displaying partial wetting.^4,25^ Parameters such as the polymer molecular weight, composition, and viscosity have been found to affect the blend morphology to some extent.^16^ However, due to the ternary nature of the systems, a dominant role is played by the interfacial tension and the equilibrium of interfacial forces between the phases, which is usually expressed by means of spreading coefficient.^11,16,29−32^ In general, the spreading coefficient for immiscible blends can be calculated as follows:
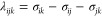
1where σ is the interfacial tension between the different polymer pairs, indicated by the subindices. Accordingly, λ*_ijk_* shows the tendency of component (*j*) to spread at the interface of component *i* and component *k*.^15,16,20^ When λ*_ijk_* is positive and the other two spreading coefficients are negative, a complete wetting morphology with phase *j* separating *i* and *k* is found (two-phase contact only). If all of the spreading coefficients are lower than zero, a partial wetting situation is encountered and the middle phase will form droplets at the interface between the two other components, giving rise to a three-phase contact line.^15,16,20^

The crystallization behavior of a given polymer can be affected by blending. In particular, a clear relationship has been found between blend morphology and crystallization of immiscible polymers since the nucleation mechanism of both the major and (especially) minor phases can be affected.^33−37^ While several researchers have studied the effect of partial/complete wetting morphology in ternary blends on their mechanical and rheological performance, to the best of our knowledge, detailed studies on the nucleation/crystallization behavior of ternary polymer blends are still missing. Some sparse information on crystallization can be extracted from the literature, revealing intriguing nucleation effects. Zolali et al. studied the compatibilization of PLA and PA11 using four different types of partially wet droplets at the interface: ethylene methyl acrylate (EMA); poly(butylene adipate-*co*-terephthalate) (PBAT); ethylene methyl acrylate-glycidyl methacrylate (EMA-EGMA); and PBS.^25^ A shift of the cold crystallization temperature of PLA to lower temperatures was recorded when PLA was in contact with EMA and EMA-EGMA droplets. In another work, Ravati et al. studied binary and ternary blends based on PLA, PBS, and PBAT.^20^ In the 33/33/33 PBS/PLA/PBAT ternary blend, displaying a complete wetting morphology, all phases crystallized coincidentally at 93 °C, suggesting an efficient nucleating effect of PLA on the other two components.

However, the crystallization of the minor component in ternary blends displaying partial wetting morphology has not yet been tackled. Hereby, we focus on this issue, considering, in particular, the nucleation behavior of PLA partially wet droplets, located at the interface with PBS and PCL phases in their immiscible ternary blend.

## 2 Materials and Methods

### 2.1 Materials

Poly(lactic acid) (PLA) (Ingeo 3001D) was purchased from NatureWorks. The polymer has a D-isomer content of around 1.4% and a weight average molar mass of 155 kg/mol. Poly(butylene succinate) (PBS) (1001MD) was purchased from Showa Denko. The weight average molar mass is equal to 60 kg/mol. Polycaprolactone (PCL) (Capa 6800), with a weight average molar mass of 87 kg/mol, was purchased from Perstorp.

### 2.2 Blend Preparation

A ternary blend comprising PCL/PLA/PBS with a composition of 45/10/45 wt % was prepared to produce a partial wetting morphology with droplets of the PLA phase located at the interface between the two major components. Prior to melt blending, the polymers were dried at 50 °C under vacuum for 24 h. The blend was prepared in an internal mixer (Brabender) equipped with roller blades. Melt mixing was performed at 190 °C and 50 rpm for 8 min under continuous nitrogen flow to prevent thermal degradation of samples. After processing, the sample was quickly quenched in ice water to freeze-in the morphology. Finally, after drying, the blend was annealed at 185 °C for 20 min under a nitrogen blanket.

### 2.3 Blend Characterization

#### 2.3.1 Scanning Electron Microscopy (SEM) Analysis

A Leica instrument (RM2165) equipped with an LN21 cooling system was used to cryogenically microtome the blend samples at −150 °C. The morphology of the sample was characterized using a desktop scanning electron microscope (SEM) at 15 kV. The BSE mode (image with backscattered electrons) was used. Selected micrographs of the most representative inner regions from different samples were obtained. The diameters of the dispersed minor phases were then measured via image analysis by counting at least 100 droplets using a Wacom digitizing table and SigmaScan v.5 software.

#### 2.3.2 Polarized Light Optical Microscopy (PLOM)

Polarized light optical microscopy (PLOM) was employed to observe the nucleation and morphology development of the PLA component in the blend. Films with a thickness of around 20–30 μm were prepared by microtoming and by gentle compression molding between two microscope glass slides on a hot plate. A polarized light optical microscope, Olympus BX51, equipped with an Olympus SC50 digital camera was used to observe spherulite development. A Linkam TP-91 hot stage was used to control the experimental temperature. PLA, PCL, and PBS were chosen due to their different crystallization and melting ranges, which allow studying the crystallization of each phase separately. The films were first held at 200 °C for 3 min to erase the effects of previous thermal history, and then they were quenched to the crystallization temperature of PLA (*T*_c_ range 120–130 °C), where the nucleation and growth of polymer spherulites were monitored. In the chosen temperature range, PLA is the only component below its melting temperature and thus able to crystallize.

#### 2.3.3 Polymer–Polymer Contact Angle

To measure the contact angle of solid PLA with molten PCL or PBS phases, thin PLA films (around 100 μm) were prepared by manual compression of PLA pellets between glass slides on a heating plate set at 200 °C, followed by their cold crystallization in an oven at 110 °C for 30 min. Then, PCL and PBS fibers were obtained by pulling small parts of molten polymer with tweezers, subsequently cut into small pieces of a few hundreds of micrometers in length. These small polymer fragments were placed on top of the solid PLA film and annealed in an oven at 125 °C for 30 min, before quenching the resulting assembly in air. This latter fast cooling stage causes the solidification of the molten polymer droplets on top of the PLA films. The contact angle is finally measured with a standard tensiometer under the assumption that the imposed thermal treatment allows to obtain and preserve an equilibrium shape of the PCL and PBS droplets wetting the PLA film. Between 10 and 20 droplets were measured for each of the two polymers.

## 3 Experimental Results

Ravati et al. examined the morphological state of ternary biodegradable polymer blends based on PLA, PCL, and PBS.^19^ A partial wetting morphology was successfully produced in all three types of ternary blends, i.e., PLA, PCL, and PBS droplets were located at the PCL/PBS, PLA/PBS, and PLA/PCL interfaces, respectively, when present as a minor component. Accordingly, Figure S1 (Supporting Information) shows a representative SEM micrograph of cryogenically microtomed surfaces of PCL/PLA/PLBS with a composition of 45/10/45 after annealing. This ternary blend displays a partial wetting morphology, where the PLA minor phase self-assembles in the form of droplets located at the interface between the co-continuous structure of the other two components (PCL and PBS).

The obtained partial wetting morphology is consistent with previous studies on the same systems.^19^ The exact shape of the droplets in the partial wetting morphology is dictated by the differences in the value of the interfacial tension between the polymer pairs, as described by the Neumann Triangle.^38^ Higher mean curvature would indicate a lower polymer–polymer interfacial tension. Thus, asymmetric PLA droplets are observed at the interface due to the substantially different PLA/PCL and PLA/PBS interfacial tensions. The average droplet size of the PLA minor phase and the percentage of the minor phase located at the interface have been evaluated via image analysis. The obtained number average and volume average diameters are 24.6 and 32.9 μm, respectively. Upon annealing, more than 98% of the minor phase is confined at the interface with a closely packed droplet morphology.

**Figure 1:**
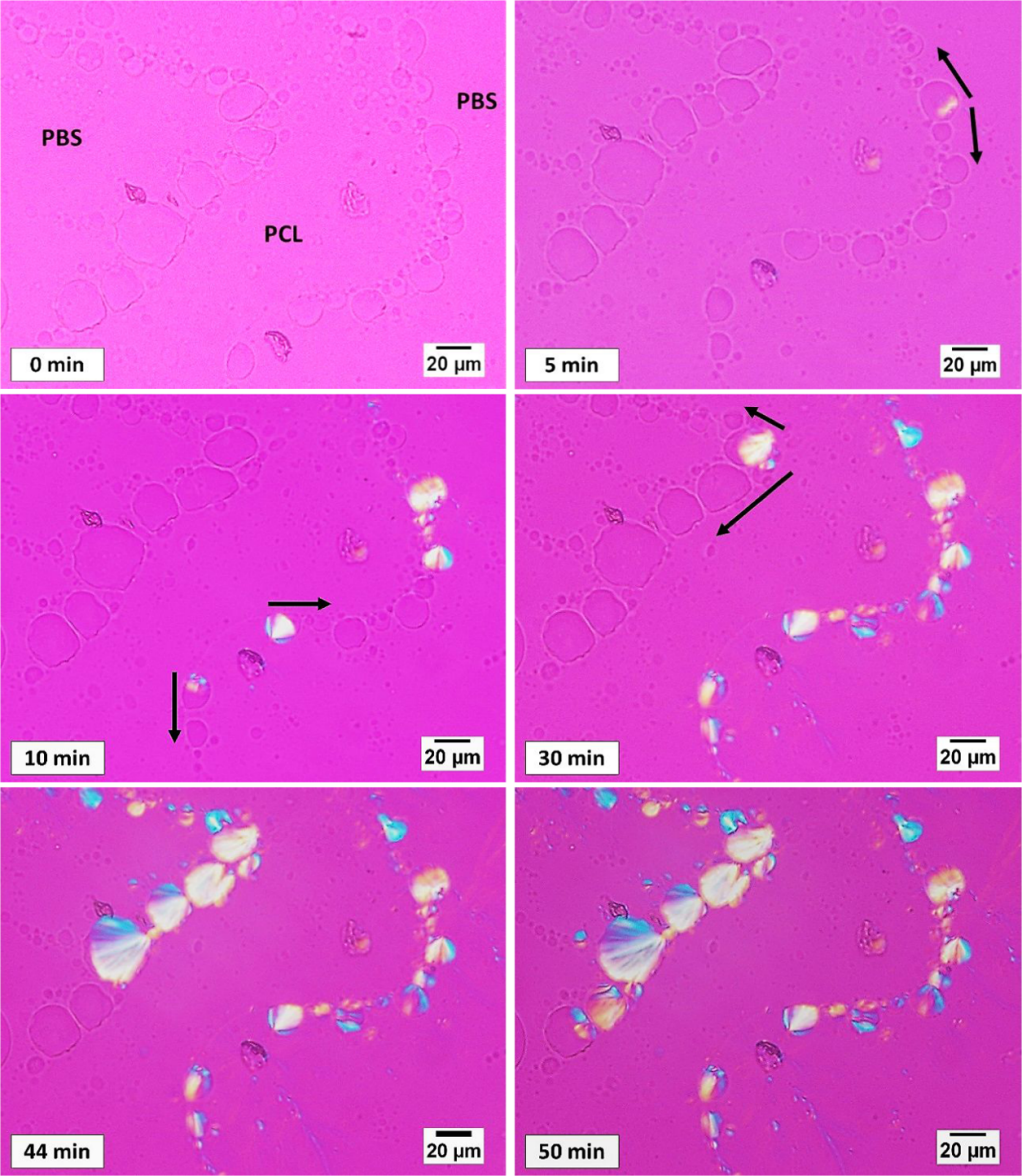
PLOM micrographs showing the crystallization of PLA droplets in a 45/10/45 PBS/PLA/PCL blend at 127.5 °C, after the indicated times. The black arrows indicate the direction of sequential crystallization.

Given the relatively large droplet size, crystallization of PLA droplets in the 45/10/45 PBS/PLA/PCL blend is particularly suitable for direct PLOM visualization, also because PLA is the polymer with the highest melting temperature in the blend, and thus it can crystallize at the interface of two molten phases, enabling maximum contrast and easy detectability of the crystalline morphologies. Figure 1 shows PLOM micrographs taken at different times during the isothermal crystallization of PLA droplets at 127.5 °C. The crystallization of PLA is initiated randomly in some of the droplets, but its progression then becomes quite directed. As indicated by the arrows in Figure 1, crystallization propagates progressively to the droplets adjacent to the initially nucleated one, in either one or two directions. Thus, nucleation spreads from one droplet to another, leading to droplet solidification in a sequential manner.

When the droplets are large enough, the morphological signature of this “sequential” nucleation is retained by the growth direction of the spherulites. It can be seen that the adjacent droplets often show a related crystal growth direction, testifying that the nucleation in the molten droplets occurs precisely from the side nearest to the neighboring crystallized droplet, where the growth of the spherulite has ended. The spread of nucleation from one crystallizing droplet to the adjacent molten one, as reported in Figure 1, can be better appreciated by visualizing the related movie, available as a Web-Enhanced Object to this article (see the Supporting Information).

The spreading of crystallization in partially wet droplets of PLA at the interface of the other polymer major phases, with nucleation occurring in molten domains adjacent to the ones that had just crystallized, resembles the “percolation” of nuclei in the crystallization of interconnected morphologies. Such phenomena are observed in co-continuous phases in immiscible blends, polymers confined in cylindrical nanopores connected by a polymer layer, and in lamellar or cylindrical microdomains in segregated block-copolymers.^33,36^ It should be noted, however, that previous studies have clearly shown that droplets of the minor phase in immiscible ternary blends displaying partial wetting form a perfectly segregated close-packed array at the interface between the two major phases.^14^ This self-assembled morphology is thermodynamically driven and “kinetically stable”. The droplets are not in contact with each other, as clearly demonstrated by different morphological analyses and as dictated by the interfacial requirement of the 3-phase contact. Even at high interfacial concentrations of minor phase domains, droplets are separated from each other by a layer of a different polymer, which can be as thin as 50 nm.^14^ The peculiar droplet-to-droplet spreading of crystal nucleation will be further analyzed in detail in Section 4 of the manuscript.

Besides this novel nucleation modality in partial wetting polymer droplets outlined above, a commonly reported nucleation mechanism in immiscible blends is nucleation at the interface. Examples of this phenomenon are reported for several binary blends, such as PLA/PCL,^39,40^ poly(ethylene oxide)/poly(caprolactone) (PEO/PCL),^41^ poly(vinylidene fluoride)/poly(lactide) (PVDF/PLA),^42,43^ isotactic polypropylene/poly(methyl methacrylate) (iPP/PMMA),^44^ and PVDF/PCL.^45^ It is worth noting that heterogeneous nucleation of the crystallizing phase can occur both at solid surfaces, when the second component has been previously crystallized,^40,42,45^ and at the liquid–liquid interface, with both polymers being in the melt state.^41,44^ The present ternary blend systems offer the possibility of investigating heterogeneous nucleation at the interface in immiscible blends where the crystallizing polymer is in contact with two chemically distinct surfaces. Of particular interest is the fact that the undercooled PLA droplets are in contact with two molten polymers (PBS or PCL).

**Figure 2:**
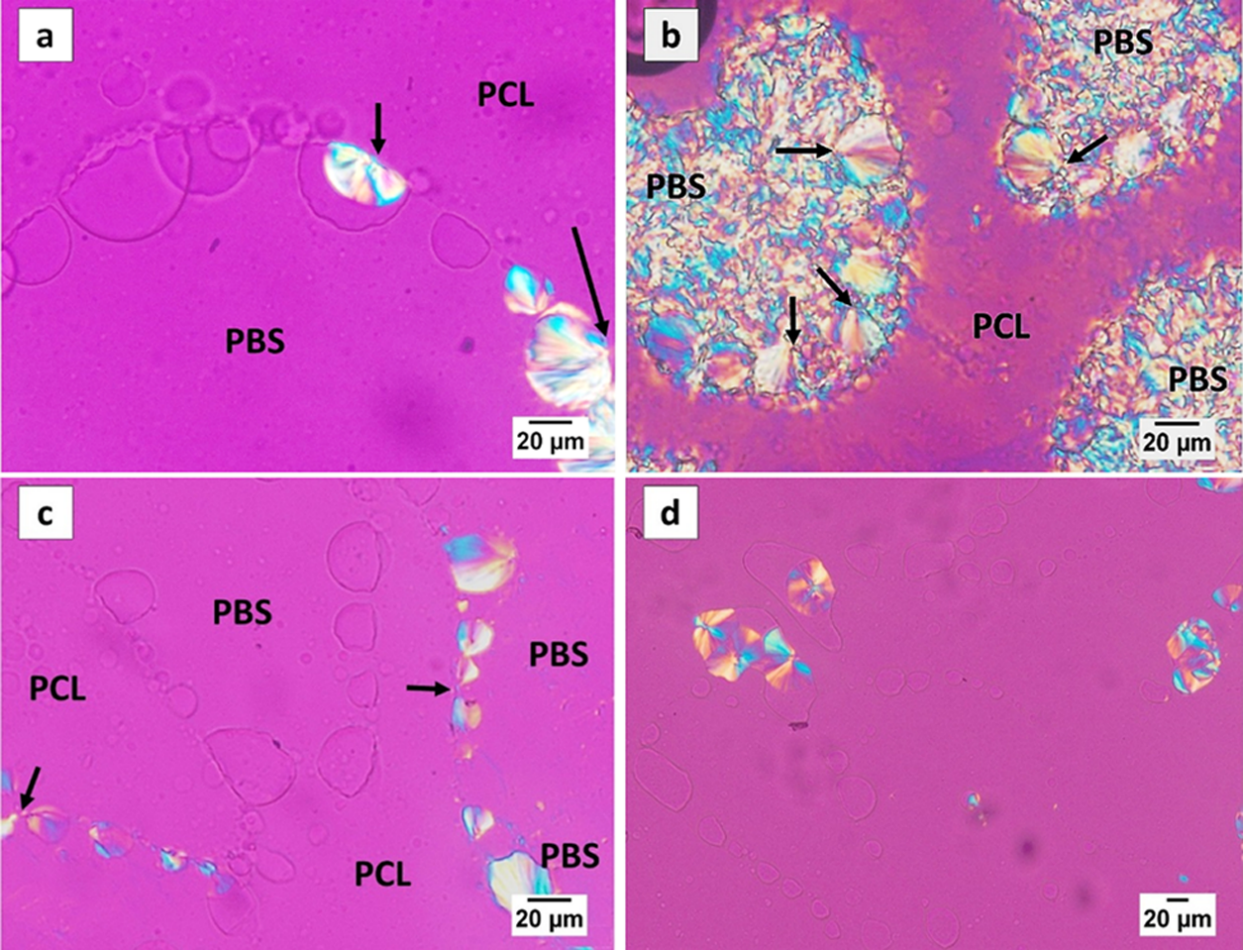
Examples of PLOM micrographs during isothermal crystallization of the PLA phase in the 45/10/45 PCL/PLA/PBS ternary blend. The pictures were taken at selected times during crystallization at (a)125 °C, (b, d) 127.5 °C, and (c) 130°C. Image (b) was captured after the occurrence of PBS crystallization at 90 °C.

Figure 2a–d shows some selected PLOM micrographs captured during the isothermal crystallization of PLA droplets. Several different nucleation modalities can be identified, as highlighted by the arrows in the images. PLA spherulites can nucleate at the interface with the molten PCL (Figure 2a), molten PBS (Figure 2b), from the three-phase contact line (a point in the PLOM transmission micrographs, Figure 2c) and eventually from the bulk of the droplet (Figure 2d).

**Figure 3:**
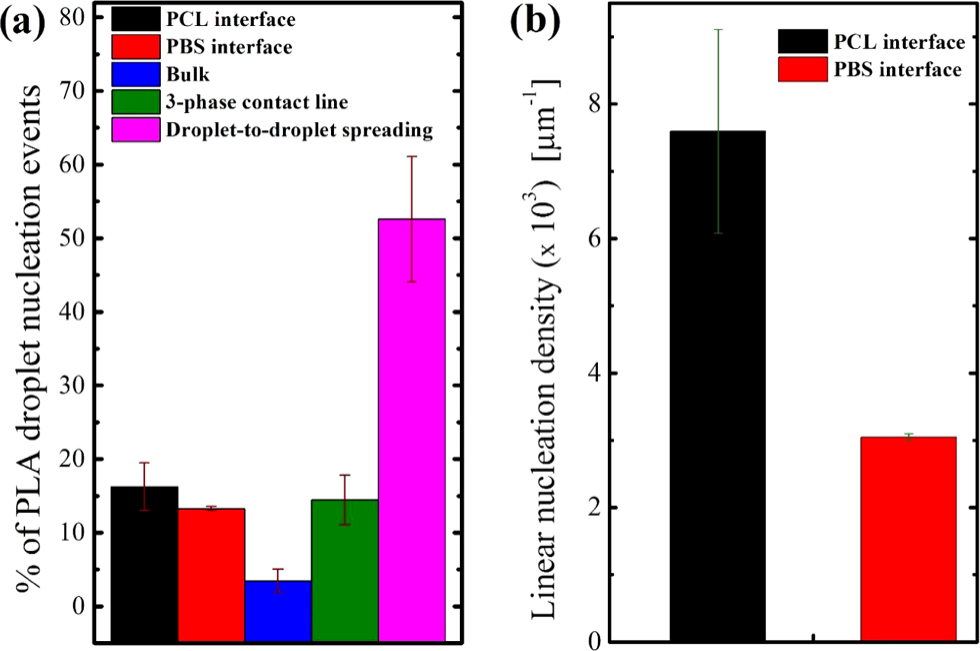
(a) Percentage of PLA droplets that nucleate according to the different modalities highlighted at a crystallization temperature *T*_c_ = 127.5 °C; (b) linear nucleation density of PLA droplets in contact with molten PCL and molten PBS phases within 45/10/45 PCL/PLA/PBS in the crystallization temperature range between 125 and 130 °C.

An attempt at quantifying the relative importance of the different nucleation modalities was made by evaluating the percentage of droplets in which solidification was initiated by each of the different mechanisms. Three different crystallization temperatures have been analyzed by considering more than 160 PLA droplets in each case from multiple samples (at least three). Figure 3a displays the results obtained for a crystallization temperature of 127.5 °C, showing the percentage of droplets nucleated from the bulk of the PLA phase, from the molten PBS or PCL interfaces, from the three-phase contact line, or by spreading of the nucleation event from previously crystallized adjacent droplets (see Figure 1). Similar data for two different crystallization temperatures are shown in Figure S2 of the Supporting Information.

Figure 3a (and Figure S2) shows that by far, the majority of the droplets nucleate according to the previously described “sequential spreading” of the nucleation event from the previously crystallized adjacent droplets. However, nucleation at one of the binary interfaces (either with PBS or PCL) or at three-phase contact line is also of importance. Nucleation in the bulk of the PLA phase is relatively less common. While the absolute values of the percentages might vary slightly with crystallization temperature (Figure S2), the overall description of the observed importance of various nucleation modalities is unchanged.

The displayed data only account for the number of droplets, without taking into account their size. To estimate if any meaningful preference exists in the nucleation of PLA at one of the two molten interfaces, it is important to consider their specific area. The droplet shape is asymmetric, and the PCL/PLA contact surface is significantly smaller than the PBS/PLA one (see Figures S1, 1, and 2). This is the consequence of the substantially higher interfacial tension between PLA/PCL (2 mN/m^28^) in comparison with that of PLA/PBS (0.2 mN/m^24^). The amount of PLA/PCL and PLA/PBS interfaces is considered by dividing the number of nucleation events occurring at each interface in the various experiments by the length of the respective contact line, calculated via image analysis software. In this way, a “linear nucleation density” is obtained, providing some hints on the different nucleation efficiencies of the two molten surfaces. The average number of PLA nuclei per micrometer of the interface is reported in Figure 3b. Given that no clear temperature dependence was found for experiments at 125–130 °C, the average data in this temperature range is presented. Despite some uncertainty in the data, the obtained results suggest that the nucleation density on the PCL interface is meaningfully larger (approximately 2 to 3 times) than that on PBS. Whether the nucleation of PLA is truly occurring at the molten immiscible polymer interface or rather on some nucleating impurities originally present in one of the polymers and transferred to a given interface during melt mixing is difficult to assess from these data. However, the possible formation of viable nuclei in contact with molten polymer surfaces will be addressed in the Discussion section.

## 4 Discussion

In this Discussion part, the spreading of the nucleation event from one crystallizing droplet to the adjacent molten one, and the nucleation of crystals of minor phase at the interface with molten polymers will be considered.

The crystallization of partially wet PLA droplets in this study clearly takes place in a sequential manner, as if some percolation exists between the different domains. How does one reconcile this with the thermodynamic definition of partially wet droplets as demonstrating points of three-phase contact? The three-phase contact unambiguously indicates that the stable partially wet droplets are separated from each other at the interface. The stability of the partially wet morphology for the PCL/PLA/PBS system is shown in Figures 1 and 2 of this study as well as in the study by Ravati et al.^18^ The theoretical determination of the wetting of this system can be estimated by calculating the spreading coefficients from the reported experimentally determined interfacial tensions between the various polymer pairs, as shown in Table 1.

**Table 1:** Experimentally Determined Values of Polymer/Polymer Interfacial Tensions, and Calculated Spreading Coefficient for the PCL/PLA/PBS Ternary Blend

interfacial tensions	spreading coefficients
γ_PBS/PLA_ = 0.20 ± 0.05 mN/m^24^	λ_PBS/PCL/PLA_ = −4.18 mN/m
γ_PBS/PCL_ = 2.38 mN/m^46^	λ_PLA/PBS/PCL_ = −0.58 mN/m
γ_PCL/PLA_ = 2.0 ± 0.7 mN/m^28^	λ_PBS/PLA/PCL_ = 0.18 mN/m

Two of the three spreading coefficients are negative, with the third one, λ_PBS/PLA/PCL_, being a small positive value close to zero. Considering the precision in the determination of γ_PLA/PCL_ (i.e., ± 0.7 mN/m^28^), these results would equally predict either very weak partial wetting or very weak complete layer formation of PLA at the PBS/PCL interface. In our work, we clearly observe partial wetting. It can thus be inferred from the above analysis that the partial wetting regime has to be very weak.

Previous work has shown that the annealing of a high concentration of partially wet droplets can sometimes result in partial to complete wetting transitions. In such a case, partially wet droplets coalesce into a completely wet layer and then proceed to dewet and return to their partially wet state.^9^ This transition is controlled by the balance between the coalescence of droplets at the interface, which tends to give a complete wetting layer, and the dewetting process, which strives to bring back the system to the most stable thermodynamic state, i.e., the partial wetting state. The speed of dewetting will be higher if the partially wet regime is “stronger”,^24^ as judged from the values of the spreading coefficients between the involved polymer pairs.

In the current study, a significant mobility of the PLA droplets at the interface can be observed during the crystallization event. Such mobility is not unexpected since crystallization is resulting in significant volume changes in the PLA droplet. These volume changes generate movement in the confined PLA droplets at the PCL/PBS interface and cause them to come into contact with one another during crystallization, as actually seen in the videos of some experiments (see Web-Enhanced Object). In turn, this likely results in coalescence and in the formation of very thin, completely wet PLA layers. When such layers are formed, although having a thermodynamic tendency to dewet, the rate for returning to the partially wet regime will be very slow due to the “weak partial wetting” conditions and the low melt temperatures. Eventually, this will lead to “quasi-stable” thin layers connecting the droplets, which then crystallize in close spatial sequence in a sequential crystallization mode, due to the existence of a percolation path.

**Figure 4:**
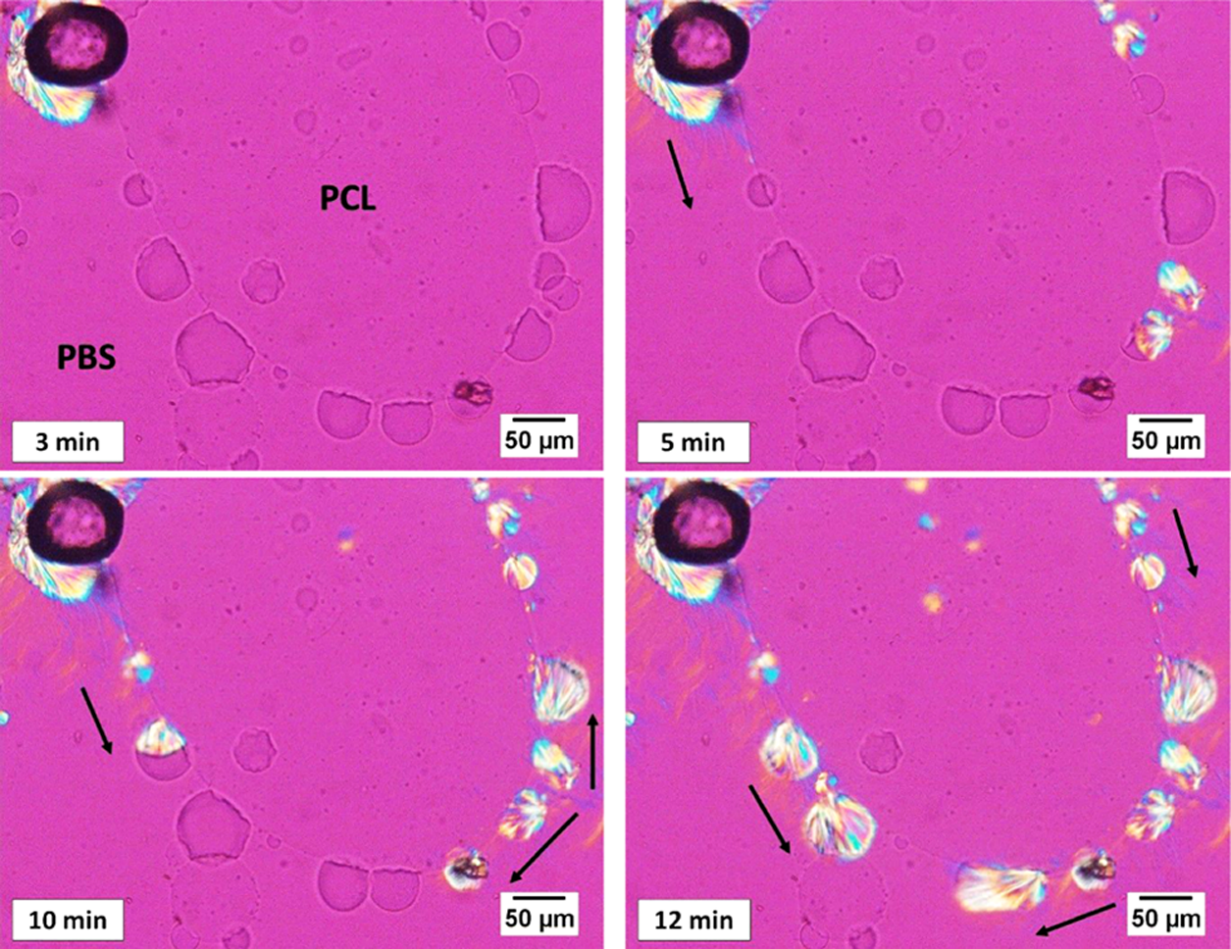
PLOM micrographs taken at the indicated times during the crystallization of PLA droplets at 130 °C in the 45/10/45 PCL/PLA/PBS blend. A faint network of thin crystalline PLA filaments, departing from a given crystallized droplet and spreading the nucleation event to the adjacent PLA domains can be seen.

**Figure 5:**
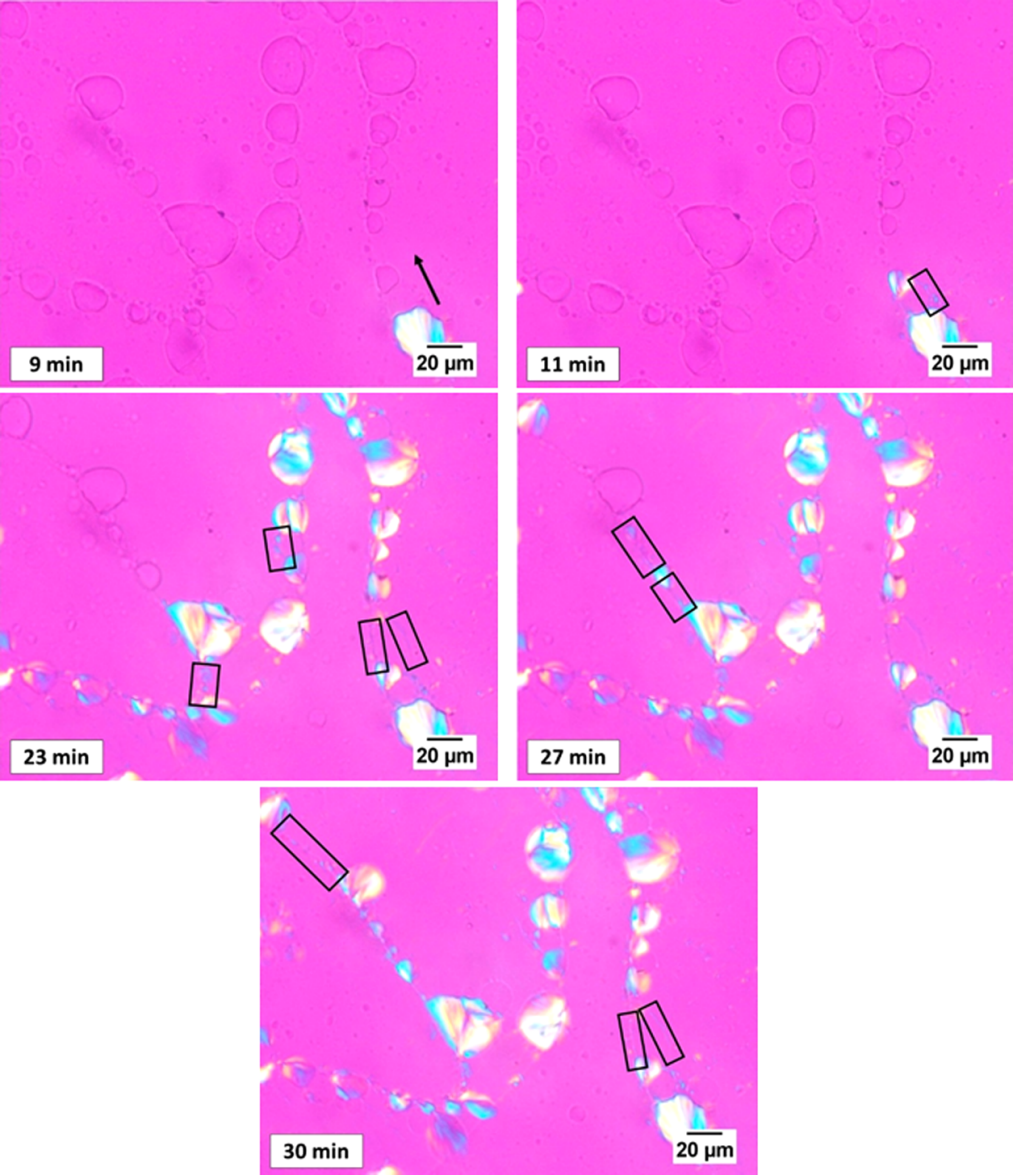
PLOM micrographs taken during the crystallization of PLA droplets at 130 °C at the indicated times in the 45/10/45 PCL/PLA/PBS blend. Rectangles show regions where thin crystalline bridges between the crystallizing larger droplets can be seen.

Some evidence supporting this mechanism is provided in Figures 4 and 5, which show two selected crystallization experiments of PLA droplets in the 45/10/45 PCL/PLA/PBS blend. In Figure 4, the PLA domains seem visually separated, in some cases, by distances of even a few tens of micrometers. Upon crystallization of a given droplet, a very faint network of crystalline fibrils develops and spreads toward the surrounding melt phases. When such branches reach the neighboring droplet, nucleation in the PLA domain suddenly occurs (see the related movie as associated Web-Enhanced Object). These PLA fibrils possibly crystallize inside the thin complete wetting layers resulting from the coalescence of the droplets, as mentioned above.

A second example is given in Figure 5. Again, the rather large PLA droplets are initially separated by large distances. During crystallization, the existence of distinctly low size droplets (or very thin continuous layers) bridging the larger domains and allowing the directional “spreading” of PLA crystallization to the adjacent droplets can be observed (see also the related video, available as Web-Enhanced Object).

As the particular droplet-to-droplet crystallization of the explored PCL/PLA/PBS ternary blend system is associated with its weak partial wetting behavior, the exploration of strong partially wet systems exhibiting lower interfacial droplet mobility would be expected to result in the crystallization of each partially wet droplet individually.

Another important observation in this work is the nucleation at the interface with molten immiscible polymer phases. Several explanations can be put forward. The most trivial interpretation of the observed nucleation at the phase boundary would be the migration of nucleating impurities, from the bulk of either phase toward the interface. However, a number of alternative interpretations, which are supported by theoretical arguments, could also justify the possible nucleation at a liquid–liquid interface in immiscible blends.

Experimental studies on an amorphous/crystalline polyolefin blend revealed enhanced nucleation of the semicrystalline component upon liquid–liquid phase separation and the results were interpreted as “fluctuation-assisted” nucleation near the interface.^47,48^ These observations can be accounted for by two theoretical frameworks.^49,50^

Using dynamic Monte Carlo simulations, Hu et al. demonstrated that immiscible binary blends exhibit weakly enhanced crystal nucleation near the interface between the two phase-separated polymers. The effect has an enthalpic origin: the equilibrium melting point of the crystal increases upon dilution of the crystallizable component by the amorphous one, and such dilution is forced to occur only at the diffuse interface.^49^ The rising of the effective melting point at the interface results in a “local” increase of the supercooling, at a given crystallization temperature, which favors nucleation. It is worth noting that, according to this theory, the enhancement of nucleation is predicted to be less pronounced for narrower or sharper interfaces due to less pronounced dilution of the crystalline component.

An alternative approach to the same problem was proposed by Muthukumar et al. In their work, they argue that spinodal decomposition causes the spontaneous formation of domains and interfaces, which can act as heterogeneous nucleation sites for the crystalline component.^50^ The combination of heterogeneous nucleation and spinodal decomposition theories allowed the authors to derive an analytical expression for the nucleation rate as a function of liquid–liquid phase separation time. The concept of heterogeneous nucleation at the liquid–liquid interfaces would equally apply when no phase separation is taking place, i.e., for immiscible blends well below their phase separation temperature (in the case of an LCST phase diagram).

**Figure 6:**
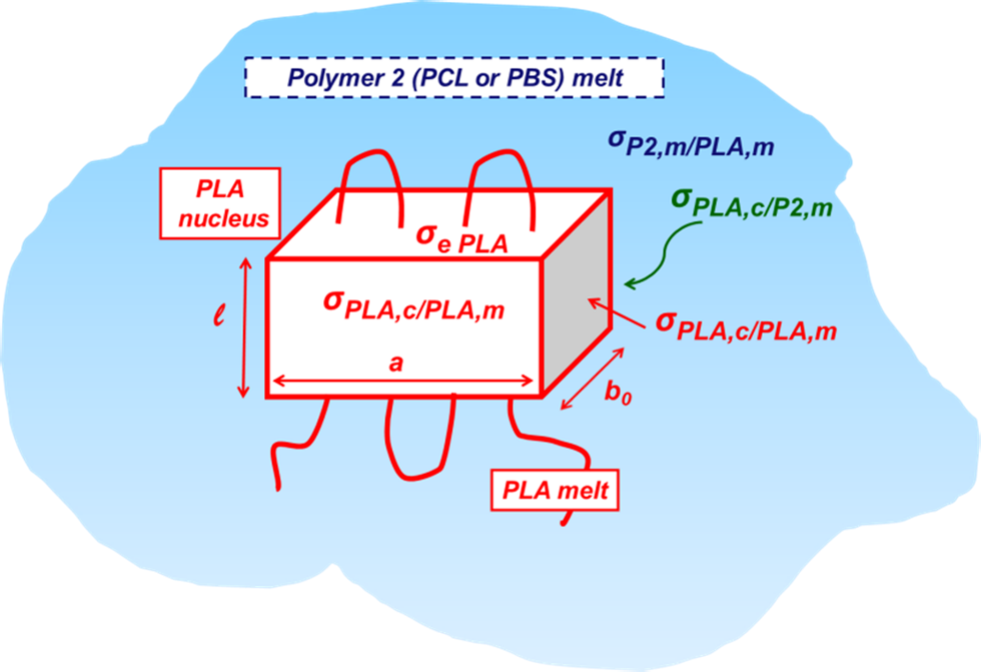
Scheme of PLA nucleus formed on a molten surface of a second polymer (Polymer 2). The dimensions and meaningful surface energies are indicated (see the text).

The applicability of the above-outlined concepts to our specific case, namely, to the apparent nucleation of PLA at the interfaces with molten PCL and PBS, will be evaluated. At first, whether the heterogeneous nucleation of PLA on a molten surface of PCL or PBS, according to the model of Muthukumar,^50^ could be a realistic option will be considered. To this aim, an estimate of the magnitude of the related free-energy barrier for nucleation will be attempted. A classical model for the heterogeneous nucleation of semicrystalline polymers on a substrate, which assumes that the free-energy barrier for nucleation is determined by the formation of the first crystalline cluster in contact with the heterogeneous surface,^51−53^ can be employed. Adapting such a model to our system, where the nucleating substrate consists of a molten polymer (PCL or PBS), the nucleus geometrical features and involved surface energies are schematically reported in Figure 6. Since the radius of curvature of the PCL/PLA and PBS/PLA interfaces is in the range of few tens of micrometers, i.e., orders of magnitude larger than the expected size of the nucleus, such an interface could be considered effectively flat.

Accordingly, the free-energy barrier for the formation of a nucleus of critical size (ΔG*) can be calculated as^51−53^
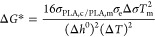
2where σ_PLA,c/PLA,m_ and σ_e_ are surface free-energies of the lateral crystal surfaces and of the chain folding surfaces, respectively, Δσ is a surface free-energy difference parameter (defined below), Δ*h*^0^ is the enthalpy of crystallization at the equilibrium melting point (*T*_m_), and Δ*T* is the supercooling degree (Δ*T = T*_m_ – *T*_c_).

The interfacial free-energy difference is a convenient way to define the nucleating ability of a substrate toward a given polymer. In essence, it represents the free-energy cost in substituting a substrate/melt interface with one crystal/substrate and one crystal/melt interface of unit area. Therefore, in the present specific case where PLA is the crystallizing polymer and the substrate is a molten surface of a second polymer (denoted as “Polymer 2” or “P2”, being either PBS or PCL), the interfacial free-energy difference would be expressed as
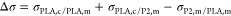
3in which *σ*_PLA,c/P2,m_ is the PLA crystal/Polymer 2 melt interfacial energy and *σ*_P2,m/PLA,m_ is the interfacial tension between the two molten polymers. Therefore, Δσ can be brought down to the surface tension properties of the polymer crystal, polymer melt, and molten blend.

The lower the value of Δσ, the more efficient is the considered nucleating substrate. To understand whether the nucleation of PLA on molten PCL or PBS is an energetically feasible option, the interfacial free-energy difference on the basis of eq 3 can be calculated. Two of the required terms, namely, the surface energy of the PLA crystals’ lateral surface and the interfacial tension between PLA and PBS (or PCL) in the melt state, can be found in the literature.

The surface tension between Polymer 2 melt and crystalline PLA can be determined by measuring the contact angle (θ) between molten PBS or PCL droplets and a solid PLA surface. In fact, by applying Young’s equation,^54^ we obtain
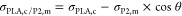
4were σ_PLA,c_ and σ_P2,m_ represent the surface tensions of PLA and Polymer 2, respectively. Samples suitable for the determination of the polymer/polymer contact angle were prepared, according to the procedure reported in Section 2. Representative examples of the micrographs employed for contact angle calculation, showing both PBS and PCL droplet profile, are reported in Figure S3 of the Supporting Information.

The results of contact angle measurements, along with the values of the required surface tensions and Δσ calculated by eq 3 are reported in Table 2. It should be noted that the results are intended as a first estimate, given the relative uncertainties in the measurements of the contact angle, on the one hand, and of the literature values of surface tension, on the other hand.

**Table 2:** Measured Polymer/Polymer Contact Angles, Estimated Surface Tensions, and Calculated Interfacial Free-Energy Difference for the Nucleation of PLA on Molten PBS and PCL Surfaces

polymer	contact angle P2,m/PLA, c [deg]^a^	polymer surface tension [mN/m]^b^	surface tension PLA,c/P2,m [mN/m]^c^	surface tension PLA,m/P2,m [mN/m]^d^	interfacial free-energy difference [mN/m]^e^
PBS	44.7	40.8	8.0	0.2	19.9 (±2.5)
PCL	45.5	39.7	9.2	2.0	19.2 (±2.5)
PLA		38.8	12.0		

a The standard deviation of the contact angles can be estimated to be ±6°.

b Values of the surface tensions are taken from ref (19) for PCL and PLA, while the value of PBS is an average between the surface tensions reported in ref (19, 55).

c These surface tensions are calculated from the measured values of the contact angle by applying eq 4. The polymer/air surface tension is calculated at 125 °C (temperature of the polymer melting treatment), using an estimated universal temperature dependence of the surface tension as σ(*T*) = σ(25 °C) – 0.06*T* (°C).^4,56^

d Values of polymer/polymer surface tensions are taken from ref (24) for PLLA/PBS and ref (28) for PLA/PCL.

e Calculated from eq 3. The precision is affected by the uncertainty of the contact angle and polymer/air surface tension.

The values of Δσ for the nucleation of PLA on molten PBS and PCL surfaces range roughly between 17 and 21 mN/m. It is useful to compare its magnitude with that of known PLA heterogeneous nucleating surfaces. Recently, we have reported a comprehensive study on the nucleation kinetics of PLA on the surface of various synthetic and natural fibers in polymer/fiber composites.^57^ The interfacial free-energy difference was derived for several substrates, from glass to carbon and hemp fibers, by measuring the nucleation rate. The obtained parameter spans from 4 to 24 mN/m, reflecting largely different nucleating activities of the fibers.^57^ As such, it can be seen that the Δσ for PLA nucleation on molten PCL or PBS, despite the approximate derivation, does not seem energetically unfavorable. Thus, the weak nucleating efficiency observed by PLOM of the ternary blend crystallization can be justified by a heterogeneous nucleation model at molten PCL and PBS interfaces, accounting for the specific surface tensions involved, without invoking any localization of nucleating impurities at the interface. This conclusion is thus consistent with earlier theoretical explanations for the nucleation of the crystallizable component in phase-separated polyolefin immiscible blends at the interface with the amorphous phase.^47,48,50^ From the values of Table 2, we can speculate about a slightly lower energy barrier for PLA nucleation on the PCL surface compared to a PBS one. This would be in agreement with the observed different nucleation tendencies of the two polymers (Figure 3b).

Another model to explain the observed nucleation of PLA on molten surfaces of PBS and PCL is the one proposed by Hu et al., which accounts for an effective increase of the equilibrium melting point at polymer/polymer interfaces, as an effect of the dilution of the crystallizable component in the interphase region of immiscible blends.^49^ We note that this theoretical interpretation predicts a stronger effect on the interfacial increase of the melting point, and hence on nucleation, for wider (i.e., more diffuse) interfaces between immiscible polymers.^49^ Interestingly, the composition across PLA/PCL and PLA/PBS interfaces in ternary blends with PLA as a minor component and with a partial wetting morphology has been measured by applying multivariate analysis using time-of-flight secondary ion mass spectroscopy.^21^ From the detected compositional gradient, a width of 2 and 3 μm was estimated for the PBS/PLA and PCL/PLA interfaces, respectively. Thus, the wider interface, which is expected to have a more marked nucleating effect,^49^ is the one between PCL and PLA. Remarkably, a higher nucleation frequency of PLA on a PCL molten interface is deduced from the reported results (Figure 3). Therefore, this correlation seems worthwhile of further investigation in the future, perhaps using ternary blends with controlled partial wetting morphology and purposely selected components giving rise to different interfacial widths.

## Conclusions

In this work, we focused our attention on the crystallization of partially wet PLA droplets at the PCL/PBS interface within ternary immiscible blends. Given the higher melting temperature, PLA crystallization can occur while the other phases remain in the molten state, and could be conveniently followed by polarized light optical microscopy observation.

A striking phenomenon is the nucleation of neighboring droplets in a sequential manner, although no continuity between the domains of the minor phase is expected in the ternary blends with partial wetting morphology. As such, the observation was explained as a consequence of the high droplet mobility at the interface, which causes the droplets to touch suddenly and promote the formation of thin complete wetting layers that interconnect them. This mechanism has been supported with experimental morphological observations.

Moreover, the interfacial nucleation of the PLA minor component on molten PCL and PBS interfaces was also shown to occur with appreciable frequency. This finding can be accounted for by a simple heterogeneous nucleation model, once the surface tension of the different polymer pairs is considered.

The achievable degree of morphological complexity in ternary blends and the interplay between morphology and nucleation will certainly require further studies. Some directions for future investigations are indicated by this first study on the crystallization of ternary polymeric blends displaying partial wetting morphology, i.e., addressing the role of interfacial tension between the molten polymer pairs in surface-induced nucleation and comparing crystallization behavior of weak and strong partial wetting droplets. The final aim is to gain a better understanding of the crystallization processes in immiscible blends to control the structure and final properties of these advanced multiphasic materials.

## Supporting Information Available

SEM micrograph of the 45/10/45 PCL/PLA/PBS blend after ultra-cryomicrotoming of the sample; percentage of PLA droplets that nucleate according to different modalities at 127.5 and 125 °C; examples of micrographs showing the contact angle of PCL and PBS droplets with PLA films (PDF)
Crystallization of partially wet PLA droplets in PCL/PLA/PBS ternary blend; example of mobility of partially wet PLA droplets at the PBS/PCL interface during PLA crystallization; crystallization of partially wet PLA droplets in PCL/PLA/PBS ternary blend (ZIP)
