# Supplementary material for: Nucleation of Poly(lactide) Partially Wet Droplets in Ternary Blends with Poly(butylene succinate) and Poly(ε-caprolactone)
Source: Macromolecules. 2020 Feb 25;53(5):1726–35. doi: 10.1021/acs.macromol.9b02295 (PMC8016171; doi:10.1021/acs.macromol.9b02295)
Supplement: Supplementary file 1 — ma9b02295_si_001.pdf [file ma9b02295_si_001.pdf]

# Nucleation of poly(lactide) partially wet droplets in ternary blends with poly(butylene succinate) and poly(ε-caprolactone)

Seif Eddine Fenni , Jun Wang , Nacerddine Haddaoui , Basil D. Favis , Alejandro J. Müller and Dario Cavallo

## SUPPLEMENTARY INFORMATION

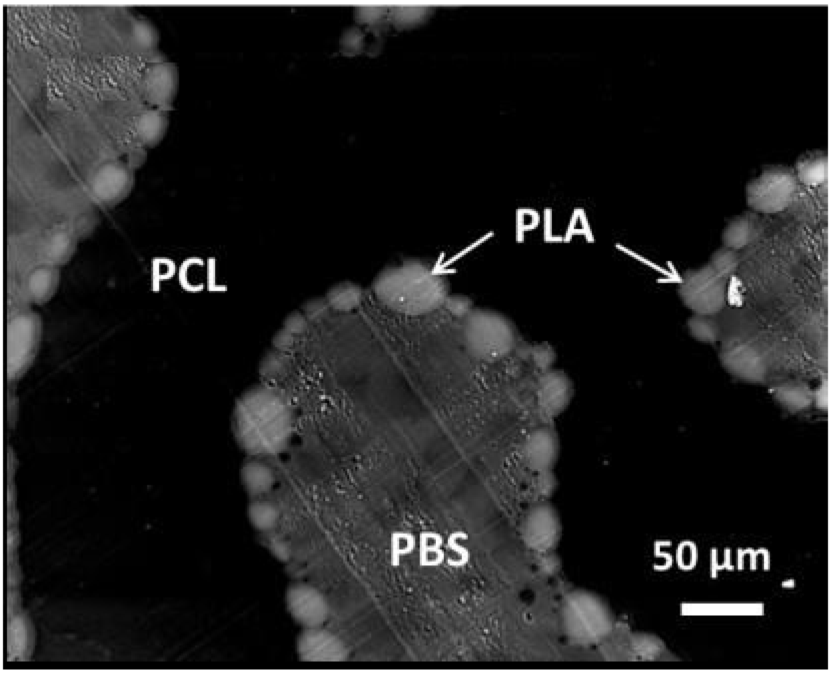


**Figure S1.** SEM micrographs of PCL/PLA/PBS ternary blend with weight composition of 45/10/45 after annealing for 20 min at 185°C.

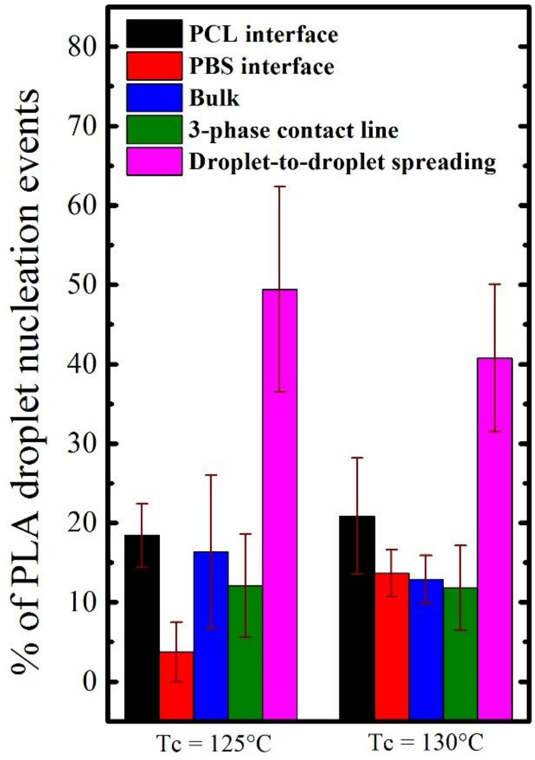


**Figure S2.** Percentage of PLA droplets that nucleate according to the different modalities highlighted, at two different crystallization temperatures $T_c$ (125°C and 130°C).

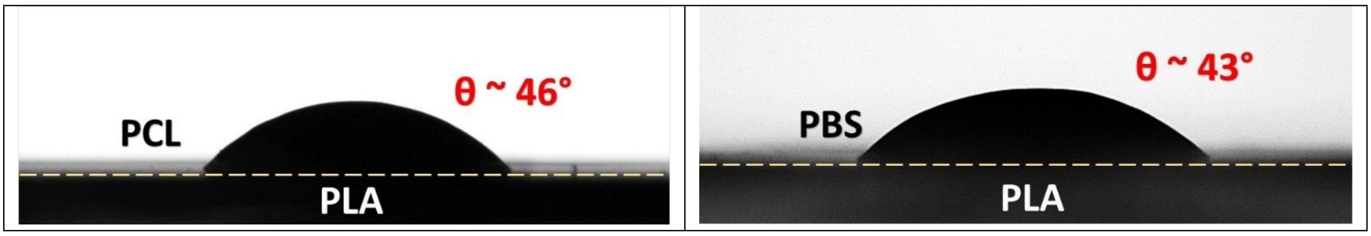


**Figure S3.** Selected photographs captured for contact angle analysis. PCL (left) and PBS (right) droplets on top of PLA film. The droplet/film interface is indicated with a dashed line.

**Web Enhanced Objects:**

**VIDEOS**

1) Crystallization of partially wet PLA droplets in PCL/PLA/PBS ternary blend (Figure 1)
2) Example of mobility of partially wet PLA droplets at the PBS/PCL interface during PLA crystallization
3) Example of mobility of partially wet PLA droplets at the PBS/PCL interface during PLA crystallization
4) Crystallization of partially wet PLA droplets in PCL/PLA/PBS ternary blend (Figure 4)

5) Crystallization of partially wet PLA droplets in PCL/PLA/PBS ternary blend (Figure 5)
